# Temperature-mediated regulation of flowering time in *Arabidopsis thaliana*

**DOI:** 10.1007/s42994-022-00069-2

**Published:** 2022-03-03

**Authors:** C. Maddie Brightbill, Sibum Sung

**Affiliations:** Department of Molecular Biosciences, The University of Texas at Austin, Austin, TX 78712 USA

**Keywords:** Floral transition, Vernalization, Thermomorphogenesis, Regulatory modules, *Arabidopsis thaliana*, Flowering pathways, Ambient temperature response, Temperature sensing

## Abstract

Throughout a plant’s life cycle, temperature plays a major role in development. Regulatory modules use temperature cues to control gene expression, facilitating physiological change from germination to flowering. These regulatory modules control morphological and molecular responses to temperature changes caused by seasonal changes or by temporary fluctuations, providing a versatile plasticity of plants. In this review, we outline how temperature changes affect the regulatory modules that induce and repress flowering, in addition to general temperature regulation. Recent studies have identified several regulatory modules by which floral transition and growth responses are controlled in a temperature-dependent manner. This review will report on recent studies related to floral transition and ambient temperature response.

## Introduction

In recent years, the sustainability of Earth has become a growing concern. Given that extreme climate events are increasing in regularity, it has become imperative to understand the effects that these changes have. Temperature shifts due to global warming and normal seasonal variations have integral effects on the distribution of plant species and on crop plant supply. Not surprisingly, temperature affects all aspects of plant development, from seed germination to flowering. Because of the increasingly fluctuating nature of modern climate and their sessile nature, understanding how plants sense and respond to temperature is more relevant than ever.

The most significant seasonal temperature change is winter cold. In certain plants, winter cold triggers the vernalization response, through which the plants acquire competence to flower rapidly in spring (Lang 1965). Ambient temperature also affects plant development and flowering in many species (Lobell and Field 2007; Long and Ort 2010). Unusually warm temperatures adversely affect plant growth and development, posing a serious threat to the food supply and plant ecosystems (Lobell and Field 2007; Long and Ort 2010). Recently, a number of regulatory modules that operate to control the growth and development of plants in response to changing temperatures have been characterized (Lin et al. 2020). It has become apparent that plants employ multiple thermo-sensory routes to reprogram their growth and development in response to temperature fluctuations (Vu et al. 2019; Lin et al. 2020). Temperature changes range from seasonal events, such as winter and spring, which influence various developmental programs in plants, to climate trends, which can result in ambient temperature fluctuation. Research pertaining to floral induction through seasonal changes has been ongoing for over several decades, while the emergence of studies relating to ambient temperature change are relatively recent. In this review, we describe recent breakthroughs in our understanding of how plant translates temperature changes to reprogram developmental transitions in plants’ life cycle with a focus on flowering.

## Temperature sensing in plants

The term “thermomorphogenesis”, used in comparison to photomorphogenesis, describes the impact of the difference in temperatures on plant morphological changes that allow the plant to adapt to temperature shifts that may otherwise be detrimental to either plant growth or development (Wigge 2013; Vu et al. 2019; Lin et al. 2020). Thermomorphogenesis relies on plant thermosensors, a term which has been generally used to describe plant components involved in sensing temperature. Plant thermosensors are defined by three criteria: (1) temperature directly impacts the thermosensor’s biochemical properties, (2) the modified properties play an important role in the signal transduction of temperature response, and (3) these changes lead to relevant changes in plant physiology or morphology (Vu et al. 2019; Lin et al. 2020). Multiple thermosensors have been identified, and it has become apparent that plants employ multiple routes to reprogram their growth and development in response to temperature fluctuations (Vu et al. 2019; Lin et al. 2020). In addition to defined thermosensors, plants employ various types of regulatory modules to sense and translate changes in temperatures into growth and developmental reprogramming.

**Fig. 1:**
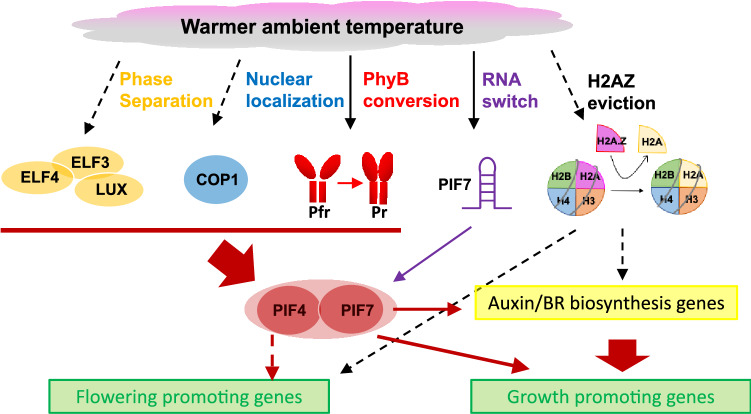
Diverse thermosensing events in plants. Plants undergo a series of biochemical changes in response to higher ambient temperature including phase separation, nuclear localization, differential kinetics of phytochrome conversion, RNA switching that alters translational efficiency, and H2AZ eviction. All changes eventually positively regulate PIF4 and PIF7 to promote growth under high temperatures. Opposite events (i.e., inhibition of growth and flowering) occur at lower ambient temperature

Physiological and morphological responses to changing temperatures include changes in flowering time, hypocotyl length, petiole elongation, leaf shape, and growth rate (Fig. 1). PIF4 and its related protein PIF7 are examples of transcription factors that mediate morphological acclimation, such as hypocotyl and petiole elongation at elevated temperatures (Franklin et al. 2011; Fiorucci et al. 2020) (Fig. 1). A red-light receptor, phyB is a thermosensor as its biochemical conversion kinetics is influenced by temperature (Jung et al. 2016; Legris et al. 2016). In addition, warm temperature facilitates the nuclear localization of COP1, a negative regulator of photomorphogenesis (Jang et al. 2015; Park et al. 2017). On the other hand, warm temperature promotes the formation of inhibitory nuclear condensates of the ELF3-containing evening complex (Jung et al. 2020). The evening complex regulates its target gene in a temperature-dependent manner in part by sequestering ELF3, which is an essential component of circadian clock, into nuclear condensates (Jung et al. 2020). These thermosensing events eventually regulate the activity of downstream transcription factors (i. e. PIF4). One of outcomes include triggering an increase in auxin biosynthesis through the transcriptional activation of *TAA1* and *CYP79B2*, which encode enzymes that function in biosynthesis of auxin-class hormones in response to higher ambient temperature (Koini et al. 2009).

The temperature-dependent nature of transcriptional regulation suggested the involvement of chromatin-level regulation. Indeed, a histone variant, H2A.Z, is involved in the regulation of global gene expression at elevated temperatures (Kumar and Wigge 2010). H2A.Z-containing nucleosomes play roles in both high temperature-induced and high temperature-repressed gene expression (Kumar and Wigge 2010; Kumar et al. 2012). Several studies have demonstrated that H2A.Z eviction as part of the transcription regulatory process takes place downstream of the thermosensors. For example, members of the INO80 chromatin remodeling complex mediates the temperature-induced removal of H2A.Z at PIF target sites, including thermoresponsive auxin-related genes (Xue et al. 2021). Another member of INO80-c, EEN, directly interacts with PIF7 and thus coordinate the H2A.Z removal as rapid responses to environmental change (Willige et al. 2021). Therefore, temperature-mediated change in chromatin landscape is a part of global changes in plants to mediate growth and developmental changes in response to temperature changes.

Although many thermosensors have recently been identified based on their aberrant behavior during thermomorphogenesis, low temperature is expected to trigger opposite events (Fig. 1). In addition, there are two additional distinct responses to near-freezing temperatures in plants, cold acclimation and vernalization. Cold acclimation mechanisms include signal transduction from the cell membrane to the nucleus through a series of biochemical and physiological changes (Thomashow 2001; Shi et al. 2018). Interestingly, components known to be involved in cold acclimation are also affected by phyB thermosensor (Jiang et al. 2020), suggesting the overlaps between cold acclimation and thermomorphogenesis. Although vernalization responds to a similar range of near freezing temperature, its response is different from cold acclimation in that it needs prolonged period of cold exposure in *Arabidopsis*. We will discuss our current understanding in mechanisms underlying temperature sensing that transduces into flowering response in more detail below. Nevertheless, alterations in temperature have an eventual role in triggering transcriptional changes in a number of genes involved in determining growth and developmental fates in plants (Fig. 1).

## Temperature effects on major regulatory modules controlling flowering time in *Arabidopsis*

Over the last several decades, comprehensive molecular genetic studies using *Arabidopsis* as a model have revealed that multiple endogenous and exogeneous stimuli affect flowering time through defined regulatory modules (Redei 1975; Koornneef et al. 1998; Kim et al. 2009; Kramer 2015; Provart et al. 2016; He et al. 2020). Two main flowering regulatory pathways that are controlled by seasonally changed environmental conditions are the photoperiod and vernalization pathways. In *Arabidopsis*, both photoperiod and vernalization pathways converge into the floral integrators. The photoperiod pathway incorporates changes in day-length and eventually controls *CONSTANS* (*CO*), which is a positive regulator of florigenic FT. The vernalization pathway is triggered by long-term cold and functions to repress FLOWERING LOCUS C (FLC), a negative regulator of FT.  Genes within flowering pathways have extensive positive and negative feedback systems, illustrating the comprehensive regulatory circuits necessary for the determination of flowering time.

Temperature affects multiple components in flowering pathways through diverse molecular events, including alternative splicing, phase separation, and temperature-dependent accumulation of regulator proteins (Vu et al. 2019; Lin et al. 2020). Both ambient growth temperature and long-term low temperature alter flowering time by modulating several floral regulators. Higher than optimal growth temperature (usually a change from 22 to 27 °C for experimental purposes) triggers accelerated flowering, even in the absence of photoperiodic cues, as an adaptation to warmer climate in *Arabidopsis* (Balasubramanian et al. 2006). In addition, a seasonal temperature change, winter cold, triggers the vernalization response, through which certain plants acquire competence to flower rapidly in spring by sensing prolonged exposure to winter cold (Lang 1965; Kim et al. 2009). Both cases result in changes in the transcription level of floral regulators, and how changes in temperature are translated into changes in gene expression has begun to unfold recently (Lee et al. 2013; Pose et al. 2013; Capovilla et al. 2015; McClung et al. 2016; Vu et al. 2019; Lin et al. 2020; Zhao et al. 2020; Zhu et al. 2021). In all cases, changes in temperature eventually results in changes in activity of floral activator, FT. Various regulatory modules are operating to sense and translate temperature changes into the activity of FT and we will discuss our current understanding on such regulatory modules below.

## Ambient temperature-mediated flowering

In *Arabidopsis*, a moderate increase in growth temperature is sufficient to accelerate flowering through the activation of FT. Over the last several years, a plethora of reports has shed light onto how a moderate change in growth temperature controls flowering time in *Arabidopsis* (Kumar and Wigge 2010; Lee et al. 2013; Pose et al. 2013; Jin and Ahn 2021). In many cases, temperature affects transcription factors which are either activators or repressors of flowering time through a florigenic FT. The balance among these floral activators and repressors determines the outcome of FT expression, which adjusts flowering in response to changes in temperature.

**Fig. 2:**
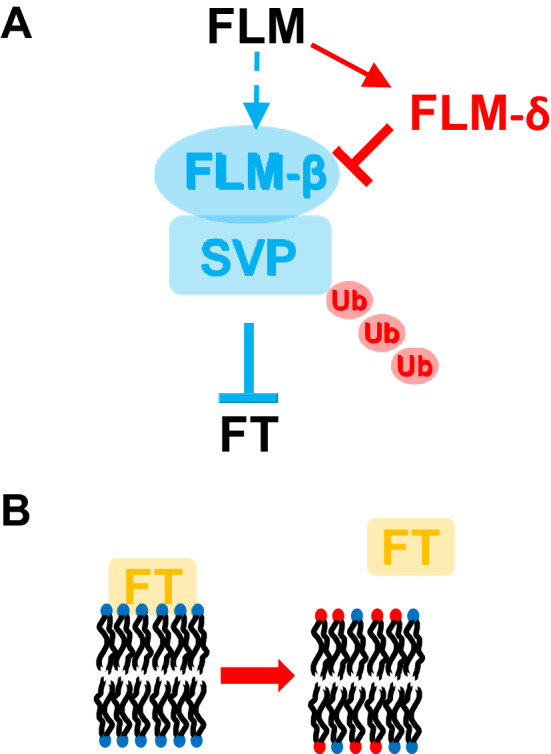
Effects on florigenic FT by ambient temperature. **A** SVP–FLM-β repressor complex represses FT. Red color indicates processes facilitated by warmer temperature, whereas blue color indicates processes facilitated by colder temperature. Warmer temperature promotes the formation of alternatively spliced transcript of FLM (FLM-δ) that acts as a dominant negative form of FLM to inhibit the SVP–FLM-β repressor complex. Higher temperature also facilitates the 26S proteosome-mediated degradation of SVP, resulting in the weakened SVP–FLM-β repressor complex at warm temperature. **B** At lower temperature, FT protein binds to membrane phospholipids and thus its mobility is limited. At higher temperature, FT is more mobile to promote the flowering

The activities of other FT regulators are subjected to temperature-mediated control through various regulatory routes (Fig. 2A). FLOWERING LOCUS M (FLM) and SHORT VEGETATIVE PHASE (SVP) are repressors of FT, and their activities are regulated upon changes in ambient temperature. FLM is alternatively spliced and the FLM-β is a component of repressor complex together with SVP. At higher ambient temperature, both FLM and SVP are regulated by two distinct mechanisms (Lee et al. 2007, 2013; Pose et al. 2013). First, a dominant negative form of FLM, FLM-δ, is alternatively spliced at higher temperature (Lee et al. 2013; Pose et al. 2013). FLM-δ poisons the repressive complex and thus allows the activation of FT. At the same time, SVP protein is degraded via the 26S proteasome, further limiting the activity of SVP–FLM-β repressor complex to repress FT (Lee et al. 2007). Therefore, the majority of molecular events triggered by changes in growth temperature result in change in expression of FT (Fig. 2).

Interestingly, a recent study showed that FT protein itself is also subject to the temperature-dependent regulation (Susila et al. 2021). As a part of the florigen, the FT protein is produced in leaf cells but must travel to the shoot apical meristem to initiate flowering (Corbesier et al. 2007; Susila et al. 2021). At low temperatures, FT binds a membrane phospholipid, with the strongest interaction being with phosphatidylglycerol. This binding limits the mobility of FT protein, and thus results in the delayed flowering at low temperature. At higher temperatures, such binding is less favored, and FT can be released to mobilize into the shoot apical meristem to promote flowering. Therefore, the temperature-dependent membrane–FT protein binding kinetics function to translate changes in temperature into flowering (Fig. 2B). Taken together, multiple flowering time regulatory modules are affected by changes in temperature; it is likely that there are more regulatory modules affected by temperature to be uncovered.

## Sensing long-term cold

During winter cold, some flowering plants undergo a process known as vernalization (Chouard 1960; Sung and Amasino 2004b; Kim et al. 2009; Antoniou-Kourounioti et al. 2021). Vernalization is a response to a long-term cold to render plants competent to flower in spring. Temperature sensing in vernalization is distinct from other temperature responses, because plants need to monitor not only the cold temperature but also the duration of cold. For example, there is no apparent connection between components mediating cold acclimation and vernalization (Liu et al. 2002). In *Arabidopsis*, vernalization resulted in epigenetically induced transcriptional repression of a major floral repressor FLC (Kim and Sung 2014; Whittaker and Dean 2017; He et al. 2020; Kinoshita and Richter 2020). At least about 4 weeks of cold temperature is necessary to establish stable repression of FLC in *Arabidopsis*. The degree of the repression of *FLC* correlates with the duration of cold (Michaels and Amasino 1999; Sung and Amasino, 2004a), indicating that the level of the repression of *FLC* reflects the duration of cold.

**Fig. 3:**
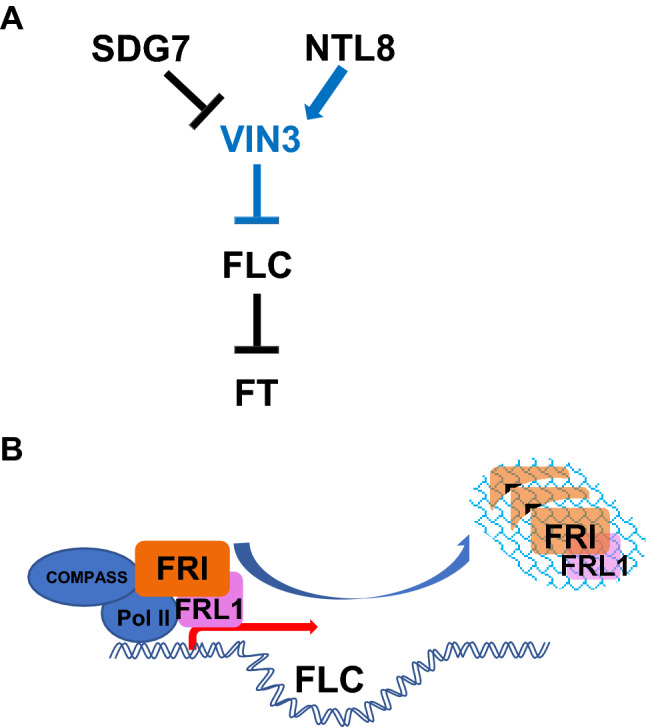
Long-term cold sensing in vernalization response in *Arabidopsis*. **A** Long-term cold transcriptionally activates the expression of *VIN3*. SDG7 is a negative regulator, whereas NTL8 is a positively regulating transcription factor. The slow accumulation of NTL8 explains slow accumulation of *VIN3* transcripts in cold. **B**. FRIGIDA (FRI) and its interacting homolog FRIGIDA-LIKE 1 (FRL1) undergoes liquid–liquid phase transition to form nuclear condensates upon cold exposure. This is proposed as a part of the mechanism for plants to cope with effects of temperature fluctuations on vernalization response. Blue arrows indicate processes occurring by cold temperature and a red arrow indicates active transcription of *FLC* prior to cold exposure

A genetic screen identified the PHD finger-containing protein, VERNALIZATION INSENSITIVE 3 (VIN3), which is required for the vernalization-mediated repression of FLC along with the PHD finger-containing domain VIL1 (Sung and Amasino 2004b). Unlike VIL1, VIN3 is induced by cold and the induction kinetics of VIN3 negatively correlates with the repression kinetics of FLC, suggesting that the VIN3 induction is controlled by mechanism that can measure the duration of cold (Sung and Amasino 2004b; Kim et al. 2010a, b). Up to date, genetic screens using VIN3-reporter transgenes have identified two putative regulators of VIN3 (Lee et al. 2015; Zhao et al. 2020). *SDG7* encodes a cytosolic protein methyltransferase that appear to repress the *VIN3* transcription prior to the cold exposure, ensuring the cold-specific induction of *VIN3* (Lee et al. 2015) (Fig. 3A). However, how the cold overcomes the repressive activity of SDG7 to induce *VIN3* is not known.

NTL8 is a NAC domain containing transcription factor that accumulates by growth and temperature (Zhao et al. 2020). Constitutively active dominant alleles of *ntl8* result in the expression of *VIN3* prior to the cold exposure and NTL8 directly bind to both *VIN3* and *NTL8* chromatin to activate the transcription, suggesting a positive feedback loop. A mathematical modeling shows that the growth-dependent accumulation of NTL8 and the effect of temperature on the rate of NTL8 accumulation is a part of mechanisms to measure long-term cold to trigger *VIN3* transcription. Interestingly, NTL8 also targets *FLC* chromatin to activate antisense COOLAIR transcription, illustrating the central role of NTL8 as a cold-specific transcriptional activator (Zhao et al. 2021).

As shown for the case of NTL8, the kinetics of *FLC* repression itself could be the result of cold temperature. Another mechanism that senses temperature change during vernalization utilizes the formation of nuclear condensates through the liquid–liquid phase separation of FRIGIDA-containing complex by cold temperature (Zhu et al. 2021). FRIGIDA is necessary for the transcriptional activation of *FLC* in part through the binding with components of a histone H3K4 methyltransferase, COMPASS-like complex, in addition to other chromatin modifiers (Li et al. 2018) (Fig. 3B). Interestingly, FRIGIDA protein accumulates more by cold when *FLC* is repressed. Increased FRIGIDA protein forms nuclear condensates with its close homologs and COOLAIR transcripts (Zhu et al. 2021). This is an intriguing mechanism to sequester a regulatory protein when it needs to be removed from the target chromatin but to store it for a possible future use. This may be an adaptation to nature environment, where temperature is expected be fluctuating and thus the formation of nuclear condensates itself serves as temperature sensor. Similar phenomenon has been observed for ELF3-containing nuclear condensates whose formation also inhibits its activity at higher temperatures (Jung et al. 2020). It remains to be shown that whether the temperature-dependent formation of such nuclear condensates is controlled by temperature-specific regulators.

## Conclusion

This review outlines how temperature affects the regulatory modules of plants to sense long-term cold and ambient temperature to mediate growth and developmental responses. Transitions between winter cold, ambient temperatures, and warm ambient temperatures each facilitate morphological and molecular responses in plants through changes in gene expression. It has become clear that temperature affects plant development through multiple regulatory modules. How plants adapt to utilize multifaceted regulatory modules to coordinate growth and developmental reprogramming is a fascinating question, especially when faced with unprecedented climate change. Recent advances in our knowledge surrounding the mechanistic details of temperature sensing and eventual changes in gene expression illustrate molecular plasticity of plants in response to temperature change, which will be essential to understanding the impact of climate change on ecosystems and agriculture.
